# The *ATO* gene family governs *Candida albicans* colonization in the dysbiotic gastrointestinal tract

**DOI:** 10.1128/mbio.01644-25

**Published:** 2025-09-03

**Authors:** Rosana Alves, Faezeh Ghasemi, Wouter Van Genechten, Stefanie Wijnants, Odessa Van Goethem, Cláudia Barata-Antunes, Vitor Fernandes, Patrícia Ataíde, Alexandra Gomes-Gonçalves, Rudy Vergauwen, Qinxi Ma, Ricardo Duarte, Isabel Soares-Silva, Margarida Casal, Alistair J. P. Brown, Patrick Van Dijck, Sandra Paiva

**Affiliations:** 1 Centre of Molecular and Environmental Biology (CBMA), Campus de Gualtar, University of Minho, Braga, Portugal; 2 Laboratory of Molecular Cell Biology, KU Leuven, Leuven, Belgium; 3 Medical Research Council Centre for Medical Mycology at the University of Exeter, Exeter, England, United Kingtom; 4 KU Leuven One Health Institute, Leuven, Belgium; Universidade de Sao Paulo, Ribeirao Preto, Sao Paulo, Brazil

**Keywords:** *Candida albicans*, short-chain fatty acids, acetate transport, AceTr family, ATO proteins, fungal infections, gastrointestinal colonization

## Abstract

The fungal pathogen *Candida albicans* colonizes the human gut, where short-chain fatty acids (SCFAs) serve as a source of carbon. This fungus harbors one of the largest microbial families of *ATO* (acetate transporter ortholog) genes, which encode putative SCFA transport proteins. Here, we generate *C. albicans* null mutants lacking individual or all known putative SCFA transporter genes and compare their phenotypes *in vitro* and *in vivo*. We show that blocking *ATO* function in *C. albicans* impairs SCFA uptake and growth, particularly on acetate. The uptake of acetate is largely dependent on a functional Ato1 (also known as Frp3/Ato3), and it is effectively abolished upon deletion of all *ATO* genes. We further demonstrate that deletion of the entire *ATO* gene family, but not inactivation of *ATO1* alone, compromises the stable colonization of *C. albicans* in the murine gastrointestinal tract following bacterial disruption by broad-spectrum antibiotics. Our data suggest that the *ATO* gene family has expanded and diversified during the evolution of *C. albicans* to promote the fitness of this fungal commensal during gut colonization, in part through SCFA utilization.

**IMPORTANCE:**

The human gut is rich in microbial fermentation products such as short-chain fatty acids (SCFAs), which serve as key nutrients for both bacteria and fungi. *C. albicans*, a common fungal resident of the gut and a cause of opportunistic infections, carries an unusually large family of *ATO* (acetate transporter ortholog) genes. This study reveals that this *ATO* gene family is required for the efficient uptake of acetate, the most abundant SCFA in the gut, and for stable colonization of the gut. These findings uncover a new layer of metabolic adaptation in fungal commensals of humans and suggest that transporter gene expansion can shape microbial fitness in response to environmental nutrient signals.

## INTRODUCTION

The mammalian gastrointestinal (GI) tract is colonized by a complex community of microbes. Microbial fermentation of dietary fibers generates short-chain fatty acids (SCFAs), which are known modulators of host-microbe interactions but also essential nutrients that support microbial growth and proliferation in the GI tract (1–3). The efficient utilization of intestinal SCFAs, either as catabolic or anabolic substrates, is promoted by specific plasma membrane transport systems.

*Candida albicans*, a human fungal pathogen that has evolved both as a commensal of the GI tract and an opportunistic pathogen, encodes transport proteins from two distinct gene families with relevance to SCFA metabolism. The *JEN* family, which includes the carboxylate transporters *JEN1* and *JEN2*, is well characterized and known to mediate carboxylate transport (4, 5). In addition, *C. albicans* has 10 genes from the evolutionarily well-conserved acetate uptake transporter (AceTr) family, also known as *ATO* (acetate transporter ortholog) genes, which have been proposed to play a role in SCFA uptake (6). This is the largest known *ATO* family in any microbe (6, 7) and the underlying drivers of the expansion of this family in *C. albicans,* together with the potential functional specialization of Ato proteins in this species, remain obscure. Indeed, acetate is the predominant SCFA in the mammalian GI tract and has long been recognized as a major carbon source for microbes (8, 9).

**Fig 1:**
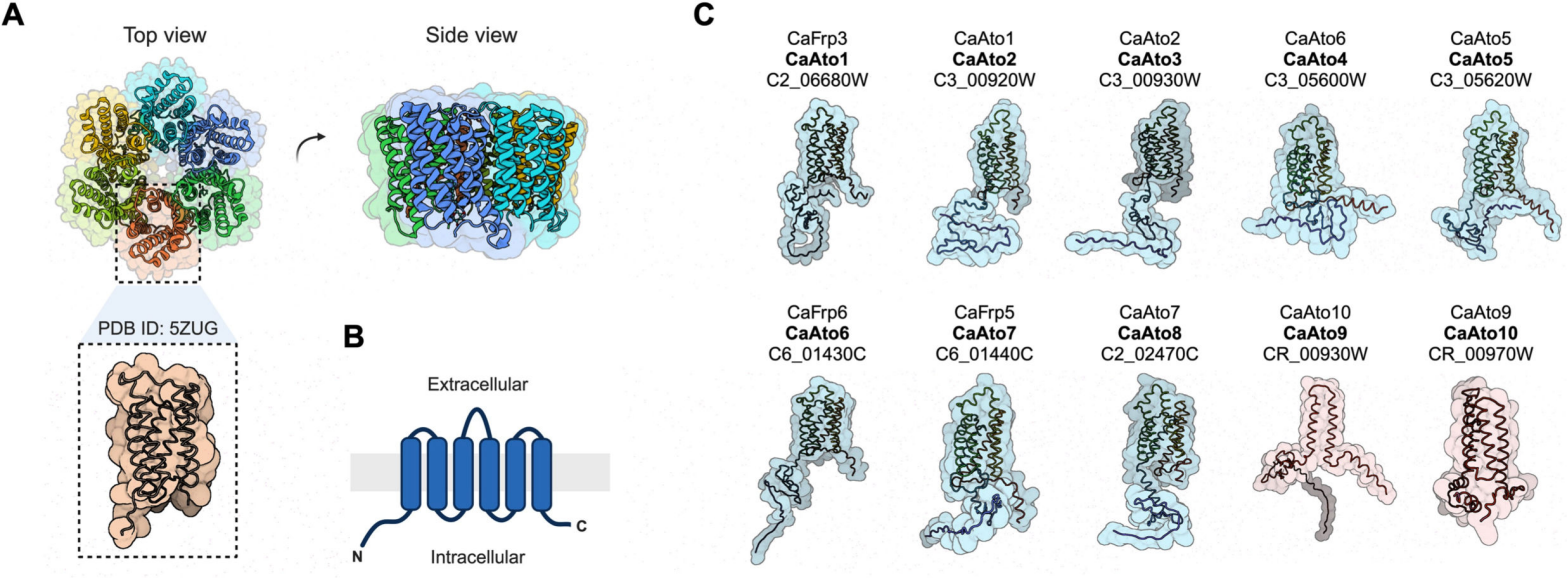
The expanded AceTr transporter family in *C. albicans*. (**A**) Top and side views of *Escherichia coli* SatP crystal structure (PDB ID: 5ZUG), a representative prokaryotic member of the acetate uptake transporter (AceTr) family. (**B**) Consensus membrane topology for each protomer (11, 12). (**C**) Schematic illustration of alpha-fold predictions for the 10 Ato membrane proteins in *C. albicans*. Displayed nomenclature follows new classification (6) (bold) to better reflect and describe their function, while maintaining reasonable consistency with the literature. Standard and systematic names are also included.

AceTr members are widely distributed across diverse taxa, including bacteria (SatP/YaaH) (10–12), archaea (AceP) (7, 13, 14), filamentous fungi (AcpA) (15, 16), and yeast (Ady2/Ato1) (6, 7, 17), where they have been reported to play a role in acetate uptake. Acetate transporters from bacterial pathogens of humans are the only AceTr members that have been structurally characterized to date. Two independently solved crystal structures have revealed that bacterial AceTr proteins form a homohexamer complex (Fig. 1A) (11, 12). Each of the six proteins consists of six transmembrane segments with their amino- and carboxy-termini located inside the cell (11, 12) (Fig. 1B). Structural and biophysical studies initially suggested a channel-like complex (11, 12), but more recent evidence has challenged this idea, indicating that acetate translocation might occur via a carrier mechanism (7, 18). Analyses of alpha-fold (19, 20) structures for the *C. albicans* Ato proteins reveal typical architectures analogous to those of bacterial AceTr family members (Fig. 1C), suggesting that Ato structural features are evolutionarily conserved from prokaryotic to eukaryotic microbes. This structural conservation supports the idea that the primary function of these membrane proteins as transporters of acetate, and potentially other SCFAs, has been retained in *C. albicans*. However, the *CaATO9* and *CaATO10* genes are most probably nonfunctional. We previously reported that these genes, which encode amino- and carboxy-terminal regions of a full-length Ato protein (Fig. 1C), appear to have evolved from a single *ATO* gene that has been split by insertion of a transposable element (6). The Ato9 and Ato10 proteins consist of only four and two transmembrane segments, respectively (Fig. 1C).

It is conceivable that an expansion of the *ATO* gene family might promote rapid adaptation of fungal cells to host-associated nutrient gradients, thereby offering clear fitness advantages in the context of human colonization or infection. Supporting this idea, mutations in certain *CaATO* genes impair the fungus’ ability to neutralize the acidic phagolysosome, a trait reported to be linked to hyphal differentiation and survival within macrophages (21, 22). However, it remains unclear whether Ato function is directly associated with virulence (23, 24). Our aim in this study was to investigate the role of the *ATO* gene family in SCFA utilization and assess its contribution to the stable colonization of the gut. We provide the first direct evidence, beyond structural inference from bacterial homologs, that Ato proteins mediate acetate transport in *C. albicans*. Moreover, we show that loss of the entire *ATO* gene family impairs stable colonization of the mammalian GI tract, highlighting the collective and environmentally contingent role of these transporters in fungal commensalism.

## RESULTS

### Inactivation of the ATO gene family in *C. albicans*

To gain further insights into the function of *C. albicans* Ato proteins, we generated a collection of single and multiple null mutants using CRISPR-Cas9 systems. Additionally, we constructed mutants lacking the monocarboxylate transporter gene *CaJEN1* and/or the dicarboxylate transporter gene *CaJEN2* to serve as controls in our assays (4, 5). Owing to the diploid nature of *C. albicans*, the lack of a traditional sexual cycle (25) and the high degree of genomic plasticity, the generation of marker-free homozygous knockout strains of multi-gene families remains technically challenging. Nevertheless, we created a set of 12 *C. albicans* mutants, each lacking a specific *JEN* or *ATO* gene, as well as strains with multiple *JEN* and *ATO* knockouts. Furthermore, we successfully eliminated all *JEN* and *ATO* alleles within a single strain.

### ATO disruption abolishes acetate utilization in *C. albicans*

**Fig 2:**
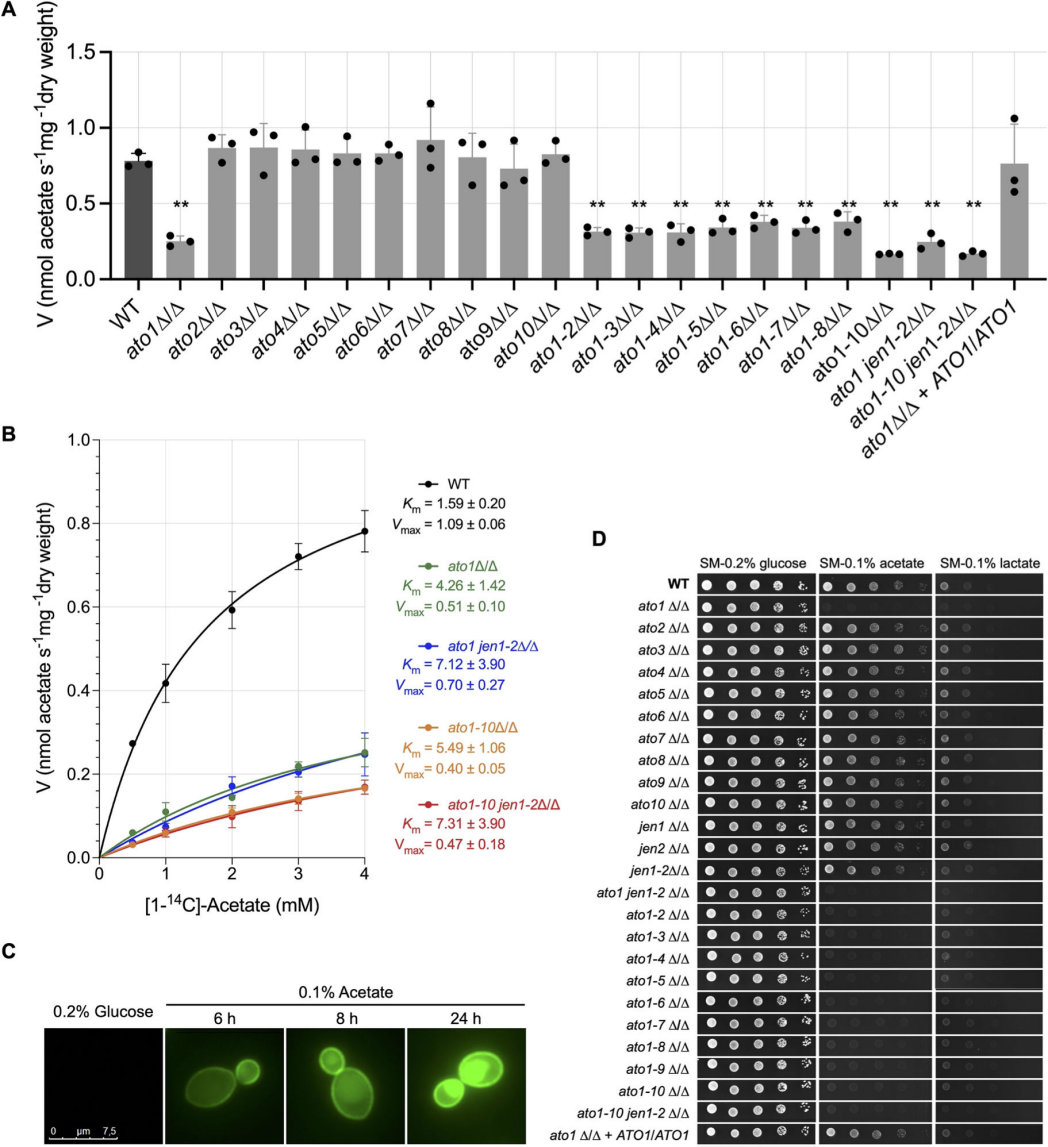
ATO disruption in *C. albicans* compromises acetate utilization. (**A**) Uptake of radiolabeled [^14^C]acetate (4.0 mM, pH 6.0) into *C. albicans* WT and knockout strains grown in Synthetic Complete (SC) medium supplemented with acetate (0.1% [vol/vol], pH 6.0). Error bars indicate mean ± SD of at least three independent experiments. Asterisks indicate statistical significance between WT and knockout strains, determined using one-way ANOVA with multiple comparisons tests: **, *P* < 0.01. (**B**) Uptake rates of radiolabeled [^14^C]acetate, at pH 6.0, as a function of acetate concentration, of cells grown in SC medium supplemented with acetate (0.1% [vol/vol], pH 6.0) as sole carbon source. The kinetic parameters (*V*_max_ and *K*_*m*_) were determined by a computer-assisted nonlinear regression analysis (GraphPad Prism 9.5.0). Error bars indicate mean ± SD of *n* = 3 independent experiments. (**C**) Expression and subcellular localization of Ato1-GFP through time in exponentially growing *C. albicans* cells in Synthetic Minimal (SM) medium supplemented with glucose (0.2% [wt/vol]) and then transferred to SC medium supplemented with acetate (0.1% [vol/vol], pH 6.0) as sole carbon source for 6, 8, and 24h. (**D**) Growth phenotypes of *C. albicans* WT and knockout strains in SM medium supplemented with specific defined carbon sources: glucose (0.2% [wt/vol]), acetate (0.1% [vol/vol], pH 6.0), and lactate (0.1% [vol/vol], pH 5.0). Cells were serially diluted, spotted on solid media, and incubated at 18°C for 7 days. Representative data from three independent replicate experiments are shown.

We screened these *C. albicans* mutants to reveal differential contributions of the 10 Ato and 2 Jen proteins to acetate uptake (Fig. 2A). As acetate transport via specific carriers follows Michaelis-Menten kinetics (26), using a saturating concentration of 4 mM ensured that differences in uptake rates between strains can be attributed to genetic modifications rather than suboptimal substrate availability. Wild-type (WT) control cells displayed three- to fivefold higher uptake rates than *ato1*∆/∆ and multiple Ato-deficient cells (Fig. 2A, *P* < 0.01). Furthermore, the deletion of *JEN* transporters in combination with *ATO* deletions did not alter the residual acetate uptake capacity (Fig. 2A). We then compared the kinetics of radiolabeled acetate transport in WT, *ato1*∆/∆, *ato1-10*∆/∆, *ato1jen1-2*∆/∆, and *ato1-10jen1-2*∆/∆ cells for concentrations between 0.5 and 4 mM (Fig. 2B). We confirmed that WT control cells displayed a high capacity to transport acetate (*K*_*m*_ 1.59 ± 0.20 mM and *V*_max_ 1.09 ± 0.06 nmol s^−1^ mg^−1^; Fig. 2B), whereas *ato1*∆/∆ cells showed a significant reduction in acetate uptake (*K*_*m*_ 4.26 ± 1.42 mM and *V*_max_ 0.51 ± 0.10 nmol s^−1^ mg^−1^; Fig. 2B). In the absence of *ATO* genes (*ato1-10*∆/∆ and *ato1-10jen1-2*∆/∆ strains), acetate transport was strongly reduced (Fig. 2B). The observed residual uptake of radiolabeled acetate in these two strains, which increases proportionally with the tested concentrations of acetate, likely represents passive diffusion of the acid into the cell (Fig. 2B).

These findings indicate that Ato1 plays an essential role in acetate transport across the *C. albicans* plasma membrane. This hypothesis is supported by Ato1-GFP expression and subcellular localization studies, where a clear Ato1-GFP signal was detected at the plasma membrane when cells were induced in the presence of acetate (Fig. 2C). There was a clear temporal increase in *ATO1* expression in response to acetate, in contrast to what is observed in the presence of glucose (Fig. 2C). The strong fluorescent signal in the plasma membrane observed at 24 h was also accompanied by fluorescence within the vacuole. This pattern is consistent with the well-documented intracellular trafficking and recycling of plasma membrane transporters in *C. albicans* and other fungi (27). Internalization and delivery to the vacuole often occur after prolonged exposure to substrate or as part of turnover and regulation mechanisms (27). Additionally, the growth of *ato1*∆/∆ cells on acetate as sole carbon source was completely abolished at 18°C but was restored upon *ATO1* reintroduction (Fig. 2D), reinforcing its essential role in acetate utilization. At this temperature, carboxylic acid uptake via passive diffusion is significantly reduced, making growth on carboxylic acids entirely dependent on the presence of a functional transporter (7). Despite the potential for genetic redundancy across this family, the presence of other *ATO* members in *ato1*∆/∆ cells did not compensate for the loss of *ATO1* in this growth condition. Indeed, we recently found that only the Ato1, Ato2, and Ato3 proteins are expressed at detectable levels in the plasma membrane when WT cells are grown on acetate, and neither Ato2 nor Ato3 showed compensatory upregulation when *ATO1* was deleted (28).

*ATO1* disruption also impaired cell growth in the presence of lactate, similar to the effect observed for the *JEN1* disruption (Fig. 2D). We then evaluated whether the deletion of additional *ATO* genes in *ato1*∆/∆ cells would confer additive growth defects in the presence of acetate or lactate under a more physiological temperature and growth condition (Fig. S1A). Strains carrying additional *ATO* gene deletions, particularly *ato1-10*∆/∆ and *ato1-10jen1-2*∆/∆, did not display any exacerbation of the growth defects observed for *ato1*∆/∆ cells at 37°C, either in synthetic complete (SC) or synthetic minimal (SM) media supplemented with acetate (Fig. S1A). Moreover, the growth defects observed in *ato1*∆/∆ cells were restored upon *ATO1* reintroduction (Fig. S1A). As previously observed at 18°C (Fig. 2D), the growth of *ato1*∆/∆ cells was also impaired in the presence of lactate as the sole carbon source at 37°C (Fig. S1A). However, complete growth abolishment under this condition was only observed upon disruption of *JEN1* (Fig. S1A).

### ATO transporter function promotes gut colonization during antibiotic-induced dysbiosis

**Fig 3:**
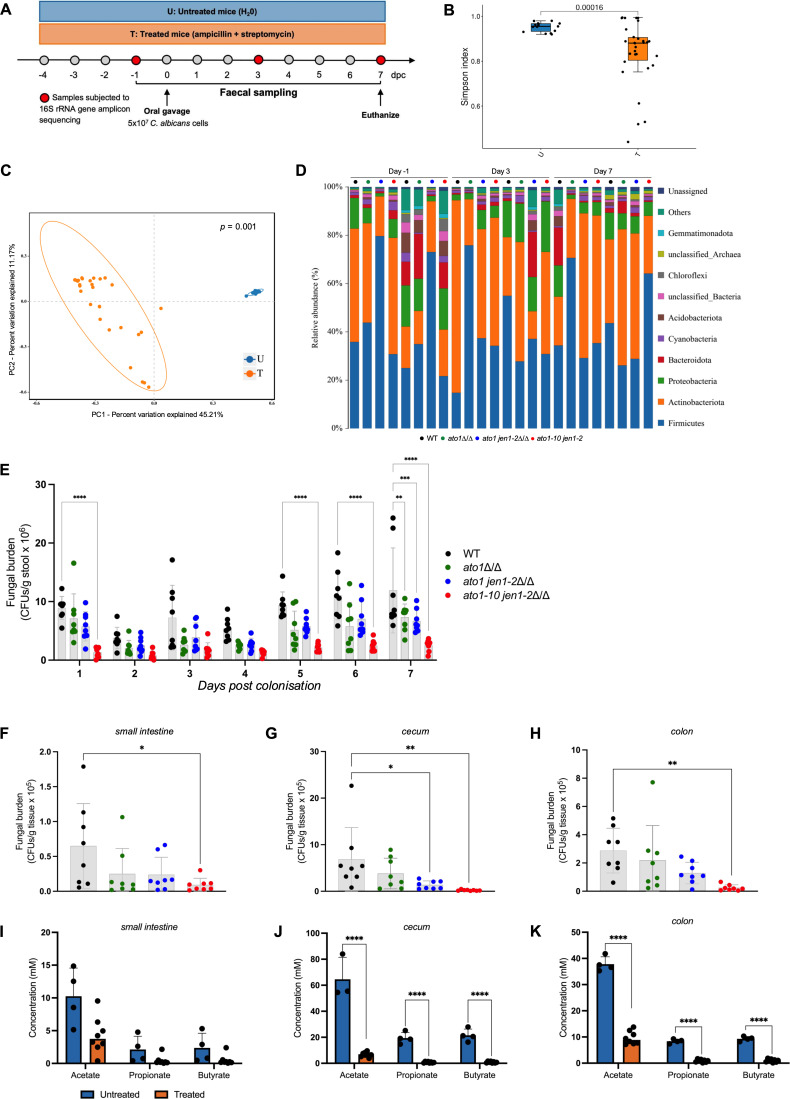
*C. albicans* ATO proteins allow stable gut occupancy of *C. albicans* under bacterial dysbiosis. (**A**) Schematic of the experimental design. Mice were colonized with *C. albicans* strains (WT, *ato1*∆/∆, *ato1 jen1-2*∆/∆, and *ato1-10 jen1-2*∆/∆) through oral gavage. Experiments were followed for 7 days post-colonization (dpc). (**B**) Alpha diversity of untreated and antibiotic-treated groups measured by the Simpson index. Data are shown via interquartile ranges with the median as a black horizontal line. (**C**) Beta diversity of untreated and antibiotic-treated groups measured by principal coordinate analysis (PCoA) based on Bray-Curtis distance. (**D**) Relative abundance of taxa (%) in antibiotic-treated mice before (day −1) and after *C. albicans* colonization through oral gavage (days 3 and 7). Each bar represents one independent group of four mice. (**E**) Fecal colonization levels (CFUs/g stool) of *C. albicans* strains (*n* = 8 per strain) in antibiotic-treated mice over time. The experiment was performed two times with similar results. Asterisks reflect comparison between strains at individual time points using Tukey’s multiple comparisons test: **, *P* < 0.01; ***, *P* < 0.001; and ****, *P* < 0.0001. (**F–H**) *C. albicans* levels (CFUs/g tissue) in the small intestine (**F**), cecum (**G**), and colon (**H**) of antibiotic-treated mice (*n* = 8 per strain) at end-point (7 days). Asterisks reflect comparison between strains at individual time points using Tukey’s multiple comparisons test: *, *P* < 0.05 and **, *P* < 0.01. (**I–K**) *in vivo* quantification of SCFAs measured in the small intestine (**I**), cecum (**J**), and colon colonized by *C. albicans* (**K**) by GC-MS. **, *P* < 0.01; ***, *P* < 0.001; and ****, *P* < 0.0001.

Altogether, these observations led us to ask whether both *ATO* and *JEN* families, or *ATO1* alone, influence fungal colonization in the GI tract—a major SCFA-rich ecological niche for *C. albicans*. To test this hypothesis, we performed a series of experiments in a murine model, monitoring the ability of *ato* and *jen* mutants to colonize the gut (Fig. 3A). The genotypes of all mutants were confirmed by whole-genome sequencing. The resident microbiota was depleted by treating mice with broad-spectrum antibiotics, a prerequisite for *C. albicans* SC5314 colonization and a well-documented risk factor for candidiasis (29). We compared the bacterial microbiotas of untreated (Fig. S2A) versus antibiotic-treated mice (Fig. 3B through D) and the *Candida*-infected and uninfected groups by 16S ribosomal RNA gene amplicon sequencing from stool samples (Fig. 3B through D; Fig. S2A). The aim of this amplicon sequencing was to examine the impact of antibiotic intervention on the intestinal microbiota and to reveal any potential changes in the gut microbiota associated with colonization by *C. albicans ato* and *jen* mutants.

The treated mice displayed decreased alpha-diversity compared with untreated controls (*P* = 0.00016; Fig. 3B), which was consistent with the antibiotic-induced depletion of bacteria. Similarly, there were significant differences in beta-diversity between untreated and antibiotic-treated samples (*P* = 0.001; Fig. 3C). Our data show that the untreated mice were mainly colonized by *Firmicutes* and *Bacteroidota* (Fig. S2A). Species of both phyla were the main targets of the administered antibiotics, with *Bacteroidota* species being partially eliminated (Fig. 3D). We also observed a significant increase in *Actinobacteriota* and *Proteobacteria* species as a result of the antibiotic treatment (Fig. 3D).

These phylogenetic alterations in the intestinal microbiome were consistent with a dysbiotic state, often observed after antibiotic treatment (30) and during colonic inflammation (31) in both murine models and clinical studies. *Candida* colonization did not alter substantially the bacterial microbiota: no significant differences were observed before and after *C. albicans* colonization (Fig. 3D; Fig. S2A). Furthermore, our comparisons of groups gavaged with *C. albicans* WT and knockout mutants showed high degrees of similarity in their microbiotas (Fig. 3D; Fig. S2A).

These analyses were important to ensure that the antibiotic treatment itself did not introduce confounding changes in bacterial community composition, independent of fungal colonization. However, these groups displayed significant differences in their fungal colonization levels (Fig. 3E). *C. albicans* levels were evaluated by quantifying fungal colony-forming units (CFUs) in feces over the course of 7 days. Significantly, none of the tested strains exhibited any fitness defects *in vitro* in the presence of glucose, either in solid (at 18°C and 37°C; Fig. 2D; Fig. S1A) or liquid media (at 30°C; Fig. S1B and C).

As expected (32), control mice that were not treated with antibiotics were not stably colonized by *C. albicans* ( Fig. S2B). In contrast, all tested strains were able to colonize the GI tract of the antibiotic-treated mice (Fig. 3E). However, under these conditions, WT cells exhibited increased fitness when compared to *ato1-10jen1-2*∆/∆ cells, with significant differences arising after five days of colonization (*P* < 0.0001; Fig. 3E). Consistent with this result, *ato1-10jen1-2*∆/∆ colonization levels were also reduced, in comparison to WT, when directly assessed in different gut compartments such as in the small intestine (*P* < 0.01; Fig. 3F), cecum (*P* < 0.01; Fig. 3G), and colon (*P* < 0.01; Fig. 3H). Fungal burdens were consistently lower in the small intestine (Fig. 3F) than in the cecum (Fig. 3G) or colon (Fig. 3H), which is in agreement with previous observations (33). The significant reduction in the colonization levels observed for the *C. albicans ato1-10jen1-2* mutant compared to both WT and *ato1* strains (Fig. 3E through H) suggests that *ATO* gene family members other than *ATO1* promote *C. albicans* fitness during gut colonization.

We also quantified the levels of the key SCFAs acetate, propionate, and butyrate in the different gut compartments by gas chromatography-mass spectrometry (GC-MS) to ensure the physiological relevance of our experimental model. The observed levels of acetate, propionate, and butyrate remained aligned with the classical reported ratio of 3:1:1 for these acids in the gut (Fig. 3I through K) (33). SCFA levels were reduced in antibiotic-treated mice, consistent with decreased bacterial fermentation under this condition. Nevertheless, these acetate concentrations remained comparable to those tested and shown to be dependent upon Ato-mediated uptake *in vitro* (Fig. 2).

## DISCUSSION

Our study demonstrates that the fungal pathogen *C. albicans* coordinates the assimilation of the key SCFA acetate via the Ato family to facilitate growth and colonization of the host. Our data are consistent with the idea that AceTr function is retained in *C. albicans* Ato1, with additive effects on acetate utilization observed as multiple *ATO* genes are eliminated. While inactivation of *ATO1* alone impairs growth on acetate, only the deletion of the entire *ATO* gene family leads to a significant reduction in the gut colonization levels of *C. albicans*. This suggests functional redundancy and highlights the environmental dependence of *ATO* gene function. The ability to transport acetate confers a metabolic advantage to *Candida* in the GI tract, where this SCFA is abundant and serves as a major available exogenous carbon source. It also enables fungal cells to sustain metabolism and survival when thriving in the diverse and complex niches of the host. The antibiotic-treated murine model essentially reflects the increased risk of candidiasis that antibiotic treatments pose to humans. Indeed, acetate concentrations in the GI tracts of antibiotic-treated mice (Fig. 3I through K) closely mirrored those reported in human feces following broad-spectrum antibiotic therapy (34), thereby validating the physiological relevance of our model.

Antibiotic treatment reshapes the gut environment in multiple, interdependent ways beyond simply reducing SCFA pools. The depletion of commensal anaerobes alters niche availability, nutrient flux, and pH and can also modulate mucosal immune responses and epithelial barrier function (26–28). Such changes may expose novel adhesion sites and/or relieve colonization resistance imposed by bacterial competitors. By probing both single- and multi-gene *ato* and *jen* mutants under these complex conditions, our study begins to unravel how SCFA uptake pathways interface with host-microbiota interactions (35). The fact that multiple ATO family members are required for robust colonization during antibiotic-induced dysbiosis suggests that the functional diversity of this expanded transporter family is critical for fungal persistence in a dynamically changing gut ecosystem.

In conclusion, our findings suggest that, during the evolution of *C. albicans*, the *ATO* gene family has expanded and diversified to promote the fitness of this fungal commensal during gut colonization, and possibly in other mucosal niches, in part through the efficient assimilation of SCFAs. We propose that ATOs are significant molecular players in the GI colonization program of *C. albicans*. Further studies are required to define how individual Ato proteins are regulated, how they function in concert, and how their activity integrates with broader networks of nutrient sensing and host adaptation.

## MATERIALS AND METHODS

### Strains and culture conditions

All *C. albicans* strains used in this study were derived from the WT strain SC5314 and are listed in Table S1. Prototrophic strains were routinely cultured in yeast extract-peptone-dextrose (YPD) medium, while nourseothricin-resistant strains were selected on YPD supplemented with 200 µg/mL nourseothricin. For *in vitro* assays, the cells were grown at 30°C, either in SM (0.69% [wt/vol] YNB from Formedium) or SC (0.67% [wt/vol] YNB from Difco, 0.2% [wt/vol] Kaiser SC mixture) liquid media supplemented with specific defined carbon sources: glucose (0.2% or 2% [wt/vol ) or acetate (0.1% [vol/vol], pH 6.0). The culture media pH was adjusted with a NaOH solution (12M). Solid media was prepared by adding agar (1.5% [wt/vol]) to the respective liquid media.

### Generation of CRISPR-Cas9 genome-edited *C. albicans* strains

Single knockout strains were generated using a transient CRISPR-Cas9 approach, with some modifications. All sgRNA expression cassettes were amplified from pV1093 plasmid in three PCR steps using sequentially the following three pairs of primers: SNR52/F and SNR52/R_GOI; sgRNA/F_GOI and sgRNA/R; and SNR52/N and sgRNA/N, where GOI represents the gene of interest. The Cas9 expression cassette was amplified from pV1093 plasmid with CaCas9/F and CaCas9/R primers. PCR reactions for both cassettes were performed according to Min et al. (36) without any modifications. Repair templates were amplified from pV1093 plasmid using NAT_GOI_repair/F and NAT_GOI_repair/R primers. The PCR was performed in a total reaction volume of 50 µL: pV1093 plasmid (50 ng), 1 µL forward primer (10 µM), 1 µL reverse primer (10 µM), 25 µL CloneAMP mastermix (Takara), and distilled water up to 50 µL. Cycling conditions included an initial denaturation step at 98°C for 2 min, followed by 40 cycles of denaturation at 98°C for 30 s, annealing at 50°C for 30 s, and extension at 72°C for 1 min, and a final step at 72°C for 10 min. *C. albicans* cells were transformed using the classical lithium acetate transformation method (37). The three cassettes were co-transformed in a single transformation (1 µg sgRNA cassette, 3 µg Cas9 cassette, and 3 µg repair template). Positive transformants were selected in YPD supplemented with nourseothricin and confirmed by colony PCR with GOI-fwd and GOI-rv primers. Multiple-knockout strains and GFP fusions (38) were generated using the Hernday HIS-FLP system (39), with some modifications. For each strain, the specific gRNA expression cassette was obtained by cloning-free stitching PCR assembly of fragment A (amplified from pADH110 with AHO1096 and AH1098 primers) and fragment B (amplified from pADH147 with AHO1097 and Hernday-GOI primers) using AH01237 and AH01236 primers. The Cas9 cassette was obtained through digestion of pADH99 plasmid with MssI restriction enzyme. Both Cas9 and gRNA cassettes were co-transformed along with a linear DNA fragment (donor DNA) in a single transformation. The 220 bp deletion donors consisted of two 120 bp annealed oligonucleotides with homology to the upstream and downstream flanks of the targeted gene. Positive transformants were selected on YPD supplemented with nourseothricin and confirmed by colony PCR. After confirming the intended deletion, the NAT marker along with the Cas9 and gRNA expression cassettes was removed from the genome by inducing the expression of the maltose-inducible flipase (FLP) recombinase system on YP 2% maltose liquid medium overnight at 30°C. Cells were then streaked on YPD agar for single-colony isolation and screened on YPD and YPD supplemented with nourseothricin to confirm efficient removal of the CRISPR components. All mutant strains were re-checked by colony PCR and validated by sequencing. Selected strains for *in vivo* assays were confirmed by whole-genome sequencing. All oligonucleotides used in this study are listed in Table S2.

### Whole-genome sequencing

Genomic DNA samples were isolated using DNeasy UltraClean Microbial Kit (Qiagen), according to the manufacturer’s recommendations. Whole-genome sequencing was performed at Novogene (UK) using Illumina NovaSeq X Plus series (PE150), with over ~100× coverage, to validate CRISPR-edited mutants and to screen for potential off-target mutations. Raw sequencing reads, previously trimmed by the sequencing company, were checked for quality using FastQC Software v.0.12.1 (https://www.bioinformatics.babraham.ac.uk/projects/fastqc/). After reads were assembled into scaffolds using MEGAHIT v.1.2.9 (40), with default settings. Next, assembly quality was assessed using QUAST software v.5.2.0 (41), also with default parameters. Gene prediction was performed with AUGUSTUS v3.5.0, using *C. albicans* SC5314 as the training model to improve accuracy. Genes of interest were aligned against each assembled genome using BLASTn with default parameters. Sequencing data are available under NCBI BioProject ID number PRJNA1286762.

### Phenotypic assays in liquid and on solid media

Phenotypic assays were performed either on solid or liquid media. For evaluation of growth phenotypes by spot assays, cells were grown in solid YPD (2% [wt/vol]) medium. The cells were then harvested, washed twice in water, and for each strain, the optical density at 640 nm was adjusted to 1. A set of four 1:10 serial dilutions was performed, and 3 µL of each suspension was spotted into the desired media, either SM or SC. Glucose (0.2% [wt/vol]) was used as a control carbon source. Cells were inoculated at 18°C for 7 days, or at 37°C for 2 days. For evaluation of growth rates, cells were pre-grown in SC media supplemented with 0.2% glucose (wt/vol) and then transferred to fresh media with an initial optical density of 0.01 at 640 nm. Optical densities were monitored every 2 h at the same wavelength. The resulting data were processed using GraphPad Prism 9 Software. Data were log-transformed (base 10), and the exponential phase for each independent experiment was selected. The log of exponential growth equation was used for calculating growth rates.

### Radiolabeled transport assays

*Candida* cells were directly grown in SC medium containing acetate (0.1% [vol/vol], pH 6.0), collected at the optical density of 0.5 at 640 nm, washed two times in ice-cold deionized water, and resuspended in water to a final density of 20–40 mg (dry weight)/mL. For each reaction, a cell suspension of 30 µL was mixed with 60 µL of potassium phosphate buffer (100 mM, pH 6.0). After 2 min of incubation at 30°C, each reaction was started by the addition of 10 µL of radiolabeled [1-^14^C]acetic acid (sodium salt, 55.2 mCi/mmol, Perkin Elmer) at the desired concentration for 15 s. Reactions were stopped by dilution with 100 µL ice-cold acetic acid (100 mM, pH 6.0). The reaction mixtures were kept on ice, centrifuged at 13,000 rpm, washed with 1 mL ice-cold deionized water, and the final pellet was resuspended in 1 mL of scintillation fluid (Opti-phase HiSafe II). Radioactivity was measured in a Packard Tri-Carb 2200 CA liquid scintillation counter. Each reaction was prepared in duplicate, and each assay was repeated three times. The transport kinetics best fitting the experimental initial uptake rates and the kinetic parameters were determined by a computer-assisted nonlinear regression analysis (GraphPad Software, Prism 9).

### Fluorescence microscopy

*C. albicans* cells were grown at 30°C in SM medium containing glucose (0.2% [wt/vo]) until mid-exponential phase (OD_640_ = 0.5), washed two times with deionized water, and then transferred to fresh SC medium supplemented with acetate (0.1% [vol/vol], pH 6.0) for 6, 8, and 24 h. The cells were examined with a Leica Microsystems DM-5000B epifluorescence microscope with appropriate filter settings. Images were acquired with a Leica DCF350FX digital camera and processed with LAS AF Leica Microsystems software. Pictures are representative of three independent experiments.

### Murine model of GI colonization

Female C57BL6 mice (aged 8–12 weeks) were obtained from Charles River Laboratories. No statistical methods were used to predetermine group size. Each group of mice (*n* = 4) was assigned at random, housed separately in individual ventilated autoclave-sterilized cages, provided with food and water *ad libitum* and adapted to standardized environmental conditions (temperature, 23 ± 2°C; humidity, 55% ± 10%; 12 h light/dark cycles) for at least 1 week before the beginning of each experiment. Four groups of mice were provided with sterile drinking water containing 2 mg/mL streptomycin and 1 mg/mL ampicillin 4 days before exposure to *C. albicans* strains and maintained on antibiotic treatment until the end of the experiment. As a control experiment, another four groups of mice did not receive any treatment. Each experiment (except control, untreated groups) was repeated two times (*n* = 8). For experimental colonization following oral gavage, *C. albicans* strains were grown overnight in YPD medium and washed twice in sterile phosphate-buffered saline (PBS). Cell densities were adjusted with PBS and confirmed by flow cytometry-based cell counting and by plating (CFUs). Mice were gavaged with 5 × 10^7^ *C. albicans* cells and sacrificed after 7 days of exposure. Fecal samples were retrieved daily before and after *C. albicans* colonization to assay fungal burdens (CFUs) and microbiome composition (16S rRNA gene amplicon sequencing). Gut compartments (small intestine, cecum, and colon) from each mouse were harvested in PBS, weighted, homogenized with glass beads using a FastPrep instrument (MP Biomedicals), and then split into samples to determine fungal burdens (CFUs) and SCFA concentrations. For CFU counting, both fecal and tissue samples were serially diluted 10-fold, 50 µL of each dilution plated on CHROMagar, and plates grown overnight at 37°C. Fungal burdens were expressed as CFU per gram of feces or tissue, respectively.

### 16S rRNA gene amplicon sequencing

Total genomic DNA was isolated from stool samples using QIAamp PowerFecal Pro DNA Kit (Quiagen) according to the manufacturer’s instructions. The DNA concentration and purity were determined with NanoDrop 2000 UV-Vis spectrophotometer (Thermo Scientific). The hypervariable region V3–V4 of the bacterial 16S rRNA gene was amplified with primer pairs 338F: 5′- ACTCCTACGGGAGGCAGCA-3′ and 806R: 5′- GGACTACHVGGGTWTCTAAT-3′. Both the forward and reverse 16S primers were tailed with sample-specific Illumina index sequences to allow for deep sequencing. The PCR was performed in a total reaction volume of 10 µL: 5–50 ng DNA template, 0.3 µL forward primer (10 µM), 0.3 µL reverse primer (10 µM), 5 µL KOD FX Neo Buffer, 2 µL dNTP (2 mM each), 0.2 µL KOD FX Neo, and finally ddH_2_O up to 20 µL. Cycling conditions included an initial denaturation step at 95°C for 5 min, followed by 20 cycles of denaturation at 95°C for 30 s, annealing at 50°C for 30 s, and extension at 72°C for 40 s, and a final step at 72°C for 7 min. The amplified products were purified with Omega DNA Purification Kit (Omega) and quantified using Qsep-400 (BiOptic). The amplicon library was paired-end sequenced (2 × 250) on an Illumina Novaseq 6000 (Beijing Biomarker Technologies Co). The bioinformatic analysis was performed with the aid of the BMKCloud (http://www.biocloud.net/). According to the quality of single nucleotide, raw data were primarily filtered by Trimmomatic v.033 (42). Identification and removal of primer sequences was processed by Cutadapt v.1.9.1 (43). PE reads obtained from previous steps were assembled by USEARCH (44) and followed by chimera removal using UCHIME (45). The high-quality reads generated from above steps were used in the following analysis. Clean reads were conducted on feature classification to output an ASVs (amplicon sequence variants) by dada2 (46), and the ASVs counts less than 2 in all samples were filtered. Taxonomy annotation of the OTUs was performed based on the Naive Bayes classifier in QIIME2 (47) using the SILVA database (48) with a confidence threshold of 70%. Alpha diversity was calculated and displayed by the QIIME2 and R software, respectively. Beta diversity was determined to evaluate the degree of similarity of microbial communities from different samples using QIIME. Principal coordinate analysis (PCoA), heatmaps, UPGMA, and nonmetric multidimensional scalin were used to analyze the beta diversity. Furthermore, we employed linear discriminant analysis (LDA) effect size (49) to test the significant taxonomic difference among groups. A logarithmic LDA score of 4.0 was set as the threshold for discriminative features. To explore the dissimilarities of the microbiome among different factors, a redundancy analysis was performed in R using the package “vegan.” Sequencing data are available under NCBI BioProject ID number PRJNA1292122.

### SCFAs extraction and quantification

SCFAs acids were extracted from gut homogenates colonized by WT *C. albicans* (small intestine, cecum, and colon). After homogenization with glass beads using a FastPrep instrument (MP Biomedicals), samples were centrifuged at 15,000 rpm for 10 min and the supernatants were taken for analysis. For each sample, 2-ethyl butyrate was used as an internal standard at a final concentration of 5 mM. A mixture of SCFAs, 10 mM each (lactate, acetate, propionate, formate, and butyrate), was used as an external standard. SCFAs were then extracted by the addition of 0.1 mL HCl (37%) and 0.4 mL diethyl ether to 0.2 mL of each supernatant followed by 1 min of vortex mixing. After centrifugation at 3,000 × *g* for 10 min, the ether layer was removed and transferred to a separate capped vial. A further 0.4 mL diethyl ether was added to the aqueous layer and a second extraction was performed. The ether extracts were combined, and 40 µL *N*-methyl-*N*-t-butyldimethylsilyltrifluoroacetamide (MTBSTFA) was added before heating at 80°C for 20 min. SCFAs were quantified simultaneously as their tertiary butyldimethylsilyl (t-BDMS) derivatives using a gas chromatography-mass spectrometer (Trace 1300–ISQ QD equipped with a TriPlus RSH autosampler and a Restek Rxi-5ms capillary GC column 30 m × 0.25 mmID). Helium was used as a carrier gas with a flow rate of 1.4 mL/min. Injection was carried out at 250°C in split mode after 1 min and with a ratio of 1:10. The temperature was first held at 50°C for 1 min and then allowed to rise to 260°C at a rate of 50°C/min, followed by a second ramp of 2°C/min until 325°C was reached; that temperature was maintained for 3 min. The mass detector was operated in scan mode (50–600 atomic mass units), using electron impact ionization (70 eV). The temperatures of the MS transfer line and detector were 325°C and 250°C, respectively. SCFAs were identified by their retention time relative to the internal standard and specific mass spectrometric patterns. Peak areas obtained from the analysis of the external standard solution, to which internal standard had been added, were used to calculate the relative response factors for each acid with respect to the internal standard. Final concentrations were determined relative to both internal and external standards and then normalized against tissue weight.

### Statistics and reproducibility

All repeated independent experiments showed similar results. No statistical method was used to predetermine sample sizes, and no data were excluded from the analyses. Statistical analyses were performed using GraphPad Prism 9 software. All data are presented as mean ± SD unless otherwise stated. All details of the statistics, including the specific tests used for each experiment, are provided in the corresponding figure legends. All schematic representations and figures were created or assembled using BioRender (https://biorender.com).

## Data Availability

Requests for further information or for resources and reagents should be directed to and will be fulfilled by the corresponding author. All unique materials generated in this study are available from the corresponding author with a completed materials transfer agreement. Any additional information required to reanalyze the data reported in this work is also available upon request. Sequencing data isare available under NCBI BioProject IDs PRJNA1286762 and PRJNA1292122.

## SUPPLEMENTAL MATERIAL

Supplemental Material mbio.01644-25-s0001.docx Figures S1 and S2; Tables S1 and S2.

ASM does not own the copyrights to Supplemental Material that may be linked to, or accessed through, an article. The authors have granted ASM a non-exclusive, world-wide license to publish the Supplemental Material files. Please contact the corresponding author directly for reuse.
